# Thinking and Action: A Cognitive Perspective on Self-Regulation during Endurance Performance

**DOI:** 10.3389/fphys.2016.00159

**Published:** 2016-04-27

**Authors:** Noel E. Brick, Tadhg E. MacIntyre, Mark J. Campbell

**Affiliations:** ^1^Department of Physical Education and Sport Sciences, University of LimerickLimerick, Ireland; ^2^Faculty of Life and Health Sciences, School of Psychology, Ulster UniversityDerry, UK; ^3^Health Research Institute, University of LimerickLimerick, Ireland

**Keywords:** self-regulation, pacing, endurance performance, attentional focus, cognitive control, metacognition, cycling

## Abstract

Self-regulation reflects an individual's efforts to bring behavior and thinking into line with often consciously desired goals. During endurance activity, self-regulation requires an athlete to balance their speed or power output appropriately to achieve an optimal level of performance. Considering that both behavior and thinking are core elements of self-regulation, this article provides a cognitive perspective on the processes required for effective pace-regulation during endurance performance. We also integrate this viewpoint with physiological and performance outcomes during activity. As such, evidence is presented to suggest that what an athlete thinks about has an important influence on effort perceptions, physiological outcomes, and, consequently, endurance performance. This article also provides an account of how an athlete might control their cognition and focus attention during an endurance event. We propose that effective cognitive control during performance requires both proactive, goal-driven processes and reactive, stimulus-driven processes. In addition, the role of metacognition—or thinking about thinking—in pace-regulation will also be considered. Metacognition is an essential component of self-regulation and its primary functions are to monitor and control the thoughts and actions required for task completion. To illustrate these processes in action, a metacognitive framework of attentional focus and cognitive control is applied to an endurance performance setting: specifically, Bradley Wiggins' successful 2015 *Hour* record attempt in cycling. Finally, future perspectives will consider the potentially deleterious effects of the sustained cognitive effort required during prolonged and strenuous endurance tasks.

## Introduction: self-regulation and endurance performance

Self-regulation has been described as change to bring thinking and behavior into accord with often consciously desired standards or goals (Forgas et al., 2009). Applied to athletic endeavor, endurance athletes must regulate speed or power output in an attempt to achieve an optimal level of performance (Foster et al., 1994; de Koning et al., 2011; de Morree and Marcora, 2013). Successful performance regularly depends on the selection of an appropriate pacing strategy, avoiding a slower-than-optimal pace and underperformance, or an over-exuberant pace during the initial stages of activity and, subsequently, premature fatigue (e.g., Abbiss and Laursen, 2008; Renfree and St Clair Gibson, 2013; Hanley, 2016). Perhaps nowhere is pacing as quintessentially *self* -regulated as during an individual time-trial, where pacing strategy is minimally influenced by other athletes or competitors, for example (e.g., Williams et al., 2015; Konings et al., 2016). One notable example is the *Hour* in cycling, an event where the performer attempts to cycle as far as possible within the allotted time. Completing the *Hour* successfully requires a consideration of numerous performance factors, including physical, nutritional, biomechanical, environmental, technological, and psychological variables (Zabala and Hopker, 2015). In 2015, Bradley Wiggins established a new ‘official’ world *Hour* record, achieving a distance of 54.526 km. Emphasizing the regulatory balancing-act required to optimally pace the *Hour*, the “non-official” world record holder, Chris Boardman (who completed a distance of 56.375 km in 1996 prior to rule changes governing the use of technology; see Zabala and Hopker, 2015), has suggested that, “In the *Hour*, you carry any mistakes with you until the end, so pacing is everything” (Wiggins, 2015, p. 13).

Much debate surrounds the processes underpinning pace-regulation during endurance activity (Abbiss et al., 2015; Renfree et al., 2015). Important recent considerations include affective state (e.g., Renfree et al., 2012; Jones et al., 2015; Rhoden et al., 2015), decision making processes (Renfree et al., 2014; Smits et al., 2014), and risk perception (Micklewright et al., 2015). However, perceived exertion has repeatedly been suggested as a key modulator of exercise intensity (e.g., de Koning et al., 2011; Eston, 2012; Smits et al., 2014) and is central to prominent models of self-paced endurance performance such as the psychobiological model (e.g., Marcora, 2010; Pageaux, 2014), and the perception-based model (Tucker, 2009). Perceived exertion has been defined as a subjective feeling of how hard or strenuous a physical task is (Borg, 1998). Despite conceptual differences on the neurophysiologic basis of effort perception and control of pacing (i.e., conscious or non-conscious; see St Clair Gibson et al., 2006; Tucker, 2009; Marcora, 2010), there is general consensus that any factor which influences perception of effort will indirectly alter pace-regulation (e.g., Marcora, 2010; Noakes, 2012). Much evidence supports this contention during endurance performance. For example, manipulation of physiological (e.g., Tucker and Noakes, 2009), pharmacological (e.g., Doherty and Smith, 2005), and environmental (e.g., competitor presence; Corbett et al., 2012; Williams et al., 2015) variables have each been shown to impact self-paced endurance performance via a dissociation of the effort perception—exercise intensity relationship.

Recent reviews of both attentional focus (Brick et al., 2014) and psychological determinants of whole-body endurance performance (McCormick et al., 2015) have also highlighted how each of these factors impact on effort perception and pace-regulation. Given that self-regulation requires both behavior (e.g., pacing) and thinking (e.g., attention) be in-line with sought after goals (Forgas et al., 2009), an increased understanding of the cognitive processes involved is important to illuminate a discussion on the regulation of endurance performance. The aim of this article, therefore, is to present, and integrate, a cognitive perspective on pace-regulation with effort perception, physiological, and performance outcomes during endurance activity. In terms of cognitive processes, the emphasis will be on attentional strategies that have been shown to impact each of these variables. This article will also consider the role of metacognition in self-regulated endurance performance.

## Thinking and pacing: attentional focus and cognitive control

An endurance athlete's focus of attention can have a significant effect on effort perception, pace-regulation, and physiological indices of performance (Brick et al., 2014). Focusing on self-regulatory cognitions such as technique or cadence/rhythm, for example, has been shown to optimize pacing without necessarily increasing the effort perceived during endurance running (e.g., Donohue et al., 2001), race-walking (e.g., Clingman and Hilliard, 1990), rowing (e.g., Connolly and Janelle, 2003), and swimming (e.g., Couture et al., 1999) tasks. Similarly, focusing on relaxing results in an improved movement economy (i.e., reduced oxygen cost) during endurance activity (e.g., Caird et al., 1999). Not all attentional foci are beneficial to performance, however. Focusing excessively on internal bodily sensations or automated processes may exacerbate effort perceptions and negatively impact pacing (e.g., Harte and Eifert, 1995; Stanley et al., 2007) or movement economy (e.g., Schücker et al., 2014), for example. Furthermore, though distractive strategies tend to reduce effort perceptions (e.g., focusing on one's environment; Stanley et al., 2007) this may be at the expense of a slower-than-optimum pace during self-paced endurance activity (e.g., Scott et al., 1999; Connolly and Janelle, 2003).

What these studies highlight is the interaction between endurance athletes' cognitions and subsequent effort perception, physiological, and performance outcomes. Recent evidence also suggests that the most appropriate attentional strategies during performance may depend on the demands of the situation (Brick et al., 2015). For instance, during a self-paced time-trial this may be to cope with distractions, or to overcome debilitating perceptions of effort while attempting to optimize performance. As such, adopting a context-appropriate focus of attention requires both a domain-specific knowledge of cognitive strategies (e.g., MacIntyre et al., 2014) and cognitive control, or the ability to regulate thoughts and actions in accord with behavioral goals (e.g., Robertson et al., 2015; Ličen et al., 2016). According to the dual mechanisms of control framework (Braver et al., 2007), cognitive control operates via two distinct modes: proactive control and reactive control (Braver et al., 2007; Braver, 2012). Proactive control involves anticipatory, goal-oriented processing of information so that attention (e.g., focus), perception (e.g., of effort), and action (e.g., pacing) are biased in a goal-driven manner (Miller and Cohen, 2001; Braver, 2012). In contrast, reactive, or stimulus-driven cognitive control (Miller and Cohen, 2001; Corbetta and Shulman, 2002; Braver, 2012) is more automatic and transient, and reacts to urgent events or conflict by engaging control only if required (Braver et al., 2007). Accordingly, reactive cognitive control is implicated in default mode processing and is less demanding on cognitive resources (e.g., working memory), whereas proactive control is engaged in more effortful situations and places a greater demand on cognitive resources (Braver, 2012; Braver et al., 2007).

Brick et al. (2015, 2016) recently proposed that both context-dependent proactive and reactive cognitive control are initiated during endurance activity. In a study involving 3 km time-trial running, the findings on the attentional focus of participants during a self-controlled pace trial (i.e., focus on pacing, monitoring distance information, and “chunking,” or mentally breaking the distance down to smaller segments) suggested both proactive and reactive forms of control were important to pace-regulation. However, when an equivalent pacing strategy was externally-controlled by the experimenter (akin to pace-making), the most frequently reported attentional foci (i.e., relaxing, optimizing running action) suggested reactive control was the predominant form of cognitive control. Heart rate was also 2% lower in the externally-controlled condition when compared with the self-controlled pace trial, possibly as a result of the cognitive strategies engaged (Brick et al., 2016). Applying these findings to endurance performance, we propose that effective pace-regulation requires the athlete to adopt a situationally-appropriate focus of attention and mode of cognitive control. During an event such as the *Hour*, for example, the athlete receives minimal and infrequent external feedback on pacing. Accordingly, perceptions of effort may serve a vital role in pace-regulation, particularly in the early stages of the event. During the latter stages, however, when the athlete begins to fatigue, cognitive strategies become more important to overcome an ever-increasing sense of effort and maintain a target pace. In support of this contention, Chris Boardman has suggested that pacing in the *Hour* is an equation with three inter-related questions: how long to go, how hard the athlete is trying, and whether that effort sustainable? He suggests the “unnerving” answer to the latter question is “maybe” (Wiggins, 2015, p. 13). Accordingly, to achieve a desired standard the athlete must proactively adopt a focus of attention to cope with task demands in a goal-driven manner. However, when faced with an unexpected event (e.g., getting distracted, errors in pacing strategy) the endurance athlete must also reactively adapt cognition when required to optimize performance or maintain positive affect, for example (e.g., Carver and Scheier, 1998; Rhoden et al., 2015).

To conclude so far, we have presented evidence to suggest what an athlete thinks about influences effort perceptions, physiological outcomes and, consequently, endurance performance. In turn, these effects of various cognitive strategies may explain when and why an athlete will engage a particular focus. Additionally, cognitive control, or the ability to regulate thoughts and actions (e.g., Braver et al., 2007) provides an insight into an athlete's ability to align thinking with performance tasks and goals. A final consideration, however, is how an athlete controls cognition and focuses attention during endurance performance. In the following section we apply Brick et al.'s (2015) metacognitive framework of attentional focus and cognitive control to self-regulation during endurance performance.

## Thinking about thinking: metacognition and endurance performance

Metacognition has been defined as an individual's knowledge and cognitions about cognitive phenomena (Flavell, 1979) or, more simply, as “thinking about thinking” (Miller et al., 1970, p. 613). Metacognition can also reflect an individual's understanding of what they know and how to use that knowledge to regulate behavior (Bransford et al., 1999; Tomporowski et al., 2015). Metacognition not only consists of conscious goals, but also the activation of strategies (i.e., thoughts, behaviors) to achieve those goals (Flavell, 1979). It is also important to note that although self-regulation and metacognition have distinct origins in psychology, metacognition is considered an essential component of effective self-regulation (Dinsmore et al., 2008; Efklides, 2008; Tarricone, 2011). Accordingly, Dinsmore et al. (2008) highlight a “conceptual core” (p. 404) binding self-regulation and metacognition that involves efforts to monitor thoughts and actions, and activity to gain control over them. As such, this section will attempt to shed further light on how endurance athletes monitor and control the thoughts and actions required for effective pace-regulation.

Brick et al. (2015) recently proposed a metacognitive framework of attentional focus and cognitive control during endurance performance. Based on the facets of metacognition (e.g., Efklides, 2008), this model comprises two distinct processes: *metacognitive skills* and *metacognitive experiences*. Metacognitive skills include *planning* prior to performance (e.g., of cognitive strategies), *monitoring* during performance (e.g., of thinking and task completion), and *reviewing and evaluating* after performance (e.g., of cognitive strategies and task performance). Metacognitive experiences, in turn, are based predominantly on monitoring processes and include both implicit and explicit *metacognitive feelings* (e.g., feeling of difficulty) and explicit *metacognitive judgments and estimates* (e.g., judging whether a cognitive strategy is effective for its intended purpose). Relevant to this perspective, Efklides (2008), for example, suggests that metacognitive experiences such as feelings of task difficulty are crucial for the self-regulation of effort.

The most relevant metacognitive skills to the present discussion are planning and monitoring processes. Metacognitive planning may incorporate proactive goal setting, establishing a desired pacing strategy, or the selection of other cognitive strategies to implement during performance (Brick et al., 2015). Metacognitive planning may be particularly important when an athlete wishes to minimalize interference from potential distractors (Miller and Cohen, 2001; Braver et al., 2007). In contrast, metacognitive monitoring predominantly involves reactive or stimulus-driven cognitive control during task performance (Corbetta and Shulman, 2002; Braver et al., 2007). Brick et al. (2015), for example, demonstrated how elite endurance runners had, through experience, developed a means of prioritizing sensory information to optimize endurance performance. Accordingly, periodic monitoring of internal sensory (e.g., perceived exertion) and/or relevant outward environmental (e.g., split times, competitors) sources of information generate implicit or explicit metacognitive feelings that form a representation of the task. Thus, while monitoring and control can occur at an implicit, non-conscious level, conscious control is engaged when metacognitive feelings (e.g., feeling of difficulty) form a representation and awareness of the task (e.g., pace is too hard) that requires an appropriate response (see Efklides, 2008). This response may be to reactively engage a cognitive strategy to cope with situational demands (e.g., focus on task-relevant stimuli) or to adopt a more appropriate pacing strategy, for example. Once initiated, the athlete may make a more explicit metacognitive judgment (e.g., this is working to maintain pace) regarding the appropriateness of their adopted focus of attention (Brick et al., 2015). Based on the outcome of this judgment, the athlete may decide to maintain their current focus, or implement an alternative, more suitable cognitive strategy.

Metacognitive skills (e.g., planning, monitoring) and experiences (e.g., feelings, judgments) may explain how endurance athletes focus attention, control cognition, and, in turn, regulate pacing. Accordingly, we propose that an athlete's efforts to monitor and control their thoughts and actions reflect the conceptual core linking metacognition and self-regulation in an endurance performance context (Dinsmore et al., 2008). To provide greater insight into these cognitive and metacognitive processes in action, the following section will integrate the theoretical constructs of attentional focus, cognitive control, and metacognitive processes with a real-world example of self-regulated pacing during endurance performance (i.e., Bradley Wiggins' successful 2015 *Hour* record attempt).

## Thinking and action: cognitive and metacognitive processes during endurance performance

**Figure 1:**
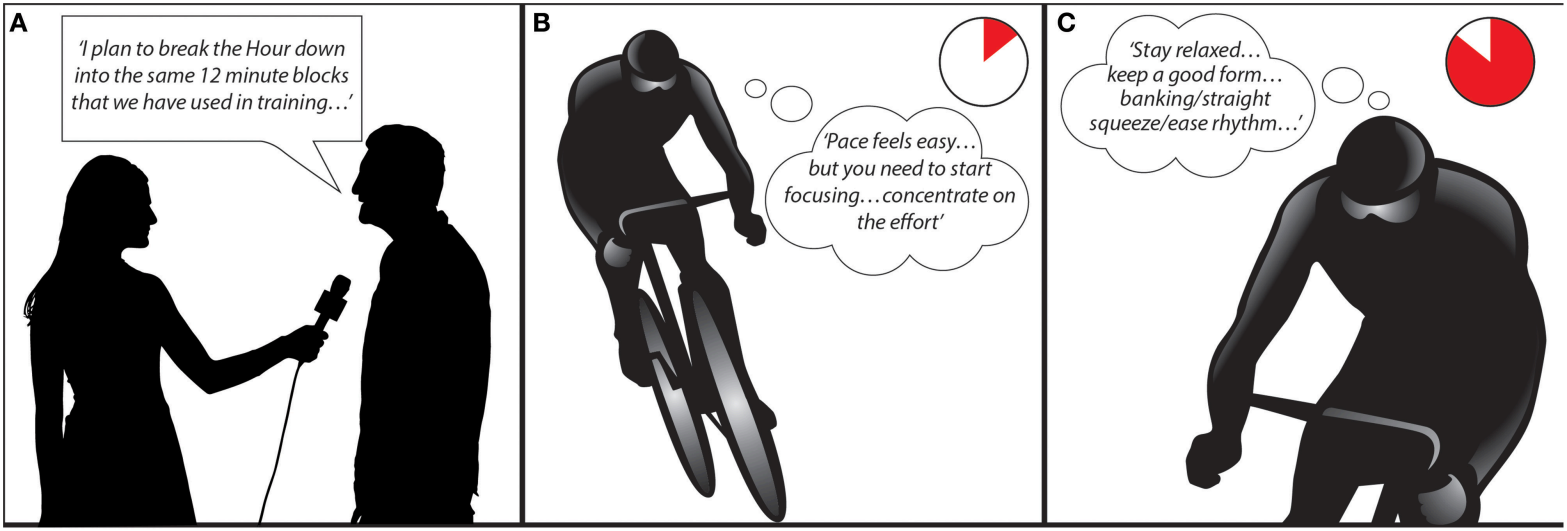
**Illustrative examples adapted from Wiggins (2015) of metacognitive planning of a cognitive strategy before performance (A), metacognitive monitoring and reactive cognitive control during performance (B), and proactive cognitive control during performance (C)**.

Many strategic considerations prior to Bradley Wiggins' 2015 *Hour* record attempt reflect metacognitive planning. His target pace (16.1 s per lap) and cadence (105 rpm) were carefully calculated to optimize his capabilities to achieve a pre-event goal distance of 55.2 km (Wiggins, 2015). One pre-planned cognitive strategy was to mentally chunk the 60 min event into blocks of 12 min, a strategy that evolved during training for the *Hour* (i.e. reflecting metacognitive planning; see Figure 1A). Although chunking as a strategy has not been investigated experimentally *per se*, reflective accounts (Brick et al., 2015, 2016) suggest that chunking may assist pace-related decision making by allowing the athlete set shorter-term goals within a longer duration endurance event.

It is also likely that the cognitive strategies Wiggins subsequently engaged during the *Hour* evolved from his 23 years' experience as a cyclist and domain-specific expertise as an elite time-trialist (Micklewright et al., 2010; Wiggins, 2012; MacIntyre et al., 2014). In this regard, evidence from his autobiographical account (Wiggins, 2015) suggests Wiggins employed both proactive and reactive cognitive control during the *Hour*. For example, during the initial stages when the pace felt easier (based on a metacognitive feeling of difficulty), he recounts self-instructions to start focusing, listening to his body, and to concentrate on the effort (i.e., reactive cognitive control; see Figure 1B). During the latter stages, however, Wiggins initiated three attentional strategies to maintain pacing and performance in a goal-driven manner (i.e., proactive cognitive control; see Figure 1C). These strategies were relaxation, focusing on form (technique), and synchronizing his pedaling rhythm with the track's banking and straight sections (Wiggins, 2015). Focusing on these active self-regulatory strategies has been shown to improve movement economy (e.g., relaxation; Caird et al., 1999), and optimize pacing without elevating effort perceptions further (e.g., technique and rhythm/cadence; Clingman and Hilliard, 1990; Connolly and Janelle, 2003). It is also noteworthy that when unexpectedly high atmospheric pressure meant his goal pace and distance may not have been attainable on the day; Wiggins recalculated his target *Hour* record pace (to 16.4 s per lap), thereby maintaining goal commitment and a positive affective state (Rhoden et al., 2015; Wiggins, 2015).

This illustrative example supports the notion that efforts to monitor and control thoughts and action link self-regulation and metacognition (Dinsmore et al., 2008; Tarricone, 2011). Furthermore, it reinforces the relationships between attentional focus, and physiologic and performance outcomes during a mentally and physically strenuous task such as an individual time-trial. As such, we suggest that further elucidation of our understanding of pace-regulation during endurance tasks will only be possible with continued integration of these scientific branches of endurance research.

## Future perspectives

The present article has highlighted the roles of attentional focus, cognitive control, and metacognition in self-regulated endurance performance. One issue worthy of further consideration concerns suggestions that inducing mental fatigue prior to activity may subsequently elevate effort perceptions and diminish endurance task performance (e.g., Marcora et al., 2009; MacMahon et al., 2014; Pageaux et al., 2015). Indeed, Marcora et al. (2009) suggest that both mentally and physically demanding tasks share the same neurocognitive resources. As such, mental fatigue may exert an influence on endurance performance by altering perceptions of effort independent of changes in cardiorespiratory or musculoenergetic mechanisms (Marcora et al., 2009). Despite these findings, no published study has specifically focused on the effects of mental fatigue accrued during sustained endurance performance. However, researchers have recently begun to speculate that prolonged endurance activity in itself may induce mental fatigue (Renfree et al., 2015; Brick et al., 2016) and reduce regulatory control (e.g., Rhoden et al., 2015). More so, while this perspective article has primarily considered pace-regulation in the context of individual time-trialing, competitive endurance events also require strategic decision-making during performance based on additional environmental factors, including competitor behavior, for example (e.g., Smits et al., 2014; Hanley, 2015; Konings et al., 2016). Given the importance of cognitive functioning to sustained endurance activity (e.g., Cona et al., 2015), deteriorations in performance during the latter stages of demanding endurance tasks may be in part attributable to increased mental fatigue and a reduced ability to maintain self-regulatory control. Further, investigation of these issues may provide a fruitful line of enquiry. It may be that additional performance gains are possible by reducing the cognitive demands associated with prolonged endurance activity. This may be achieved by adopting an appropriate focus of attention (e.g., relaxing), for example, or by utilizing pace-makers to reduce pace-related decision making during prolonged endurance events.

## Author contributions

NB: Conceptualizing and drafting the article, revising it critically for important intellectual content, final approval of the version to be published, and accountability for all aspects of the work. TM: Conceptualizing and revising the study critically for important intellectual content, final approval of the version to be published, and accountability for all aspects of the work. MC: Conceptualizing and revising the study critically for important intellectual content, final approval of the version to be published, and accountability for all aspects of the work.

### Conflict of interest statement

The authors declare that the research was conducted in the absence of any commercial or financial relationships that could be construed as a potential conflict of interest.
